# Identification of candidate genomic regions for egg yolk moisture content based on a genome-wide association study

**DOI:** 10.1186/s12864-023-09221-8

**Published:** 2023-03-14

**Authors:** Ruiqi Zhang, Fusheng Yao, Xue Cheng, Mengyuan Yang, Zhonghua Ning

**Affiliations:** Department of Animal Genetics and Breeding, National Engineering Laboratory for Animal Breeding, College of Animal Science and Technology, China Agricultural University, 100193 Beijing, China

**Keywords:** Thermogelled egg yolks, Water content, Heritability, Genome-wide association study

## Abstract

**Background:**

Eggs represent important sources of protein and are widely loved by consumers. Egg yolk taste is an important index for egg selection, and the moisture content of the egg yolk affects the taste. To understand the molecular mechanism underlying egg yolk moisture content, this study determined the phenotype and heritability of egg yolk water content and conducted a genome-wide association study (GWAS) using a mixed linear model.

**Results:**

We determined the phenotype and heritability of thermogelled egg yolk water content (TWC) and found that the average TWC was 47.73%. Moreover, significant variations occurred (41.06–57.12%), and the heritability was 0.11, which indicates medium-low heritability. Through the GWAS, 48 single nucleotide polymorphisms (SNPs) related to TWC (20 significantly, 28 suggestively) were obtained, and they were mainly located on chromosomes 10 and 13. We identified 36 candidate genes based on gene function and found that they were mainly involved in regulating fat, protein, and water content and embryonic development. *FGF9*, *PIAS1*, *FEM1B*, *NOX5*, *GLCE*, *VDAC1*, *IGFBP7*, and *THOC5* were involved in lipid formation and regulation; *AP3S2*, *GNPDA1*, *HSPA4*, *AP1B1*, *CABP7*, *EEF1D*, *SYTL3*, *PPP2CA*, *SKP1*, and *UBE2B* were involved in protein folding and hydrolysis; and *CSF2*, *SOWAHA*, *GDF9*, *FSTL4*, *RAPGEF6*, *PAQR5*, and *ZMAT5* were related to embryonic development and egg production. Moreover, *MICU2*, *ITGA11*, *WDR76*, *BLM*, *ANPEP*, *TECRL*, *EWSR1*, and *P4HA2* were related to yolk quality, while *ITGA11*, *WDR76*, *BLM*, and *ANPEP* were potentially significantly involved in egg yolk water content and thus deserve further attention and research. In addition, this study identified a 19.31–19.92 Mb genome region on GGA10, and a linkage disequilibrium analysis identified strong correlations within this region. Thus, GGA10 may represent a candidate region for TWC traits.

**Conclusion:**

The molecular genetic mechanism involved in TWC was revealed through heritability measurements and GWAS, which identified a series of SNPs, candidate genes, and candidate regions related to TWC. These results provide insights on the molecular mechanism of egg yolk moisture content and may aid in the development of new egg traits.

**Supplementary Information:**

The online version contains supplementary material available at 10.1186/s12864-023-09221-8.

## Background

Eggs are rich in fat, minerals, and vitamins and considered an excellent source of protein [1]. The internal quality of eggs is an important index used by the poultry industry to evaluate eggs [2], and associated index factors include the protein height, Hastelloy unit, yolk texture, and yolk taste. Boiling can retain the nutrients in eggs to the greatest extent compared with other cooking processes and thus facilitates the absorption of nutrients from the eggs. However, the yolk taste of boiled eggs is often criticized by consumers [3]. Consumers may find that chewed thermogelled gel yolks are difficult to swallow because in the process of chewing and swallowing, thermogelled gel yolks absorb water from the mouth and throat, resulting in a dry throat and difficulty swallowing [4]. The moisture content in thermogelled gel yolks affects the fineness of the yolk texture, with a higher the moisture content leading to a finer texture [5]. Therefore, studying the thermogelled egg yolk water content (TWC) can help resolve the problem of poor yolk taste and provide a better understanding of the yolk texture.

Egg yolk is a valuable material component not only because of its nutritional components but also because of its sensory characteristics [6]. The complex composition of egg yolks endows these components with a unique texture and flavor, thereby promoting their wide use in food processing. During heat treatment, egg yolks become folded into a three-dimensional network structure and undergo irreversible deformation, changing from liquid to solid, which leads to the characteristic texture [7]. The substances in egg yolk play an important role in this process. The protein structure is destroyed under high temperature, which changes the spatial structure of yolk. Fat particles act as active fillers in the protein gel network based on interactions between proteins in the network [8]. Water is wrapped by proteins and lipids, which affects the fineness of the texture [9]. The water content in egg yolk is not only affected by the protein and lipid contents and environment of the egg but also by genetic factors [10]. However, the effects of genetic factors on egg yolk water content have not been previously studied.

Heritability can be used to evaluate the proportion of traits controlled by genetic factors. The higher the heritability, the greater the proportion of genetic factors and the smaller the proportion of environmental factors involved in a trait. Determining heritability can provide insights on the genetic mechanisms underlying traits. Whole genome sequencing is a sensitive, fast, and accurate technology [11]. Genome wide association analysis (GWAS) is a statistical tool and one of the effective methods of identifying important single nucleotide polymorphisms (SNPs) and candidate gene [12]. Compared with genotyping chips, the genes covered by high-throughput sequencing are more comprehensive. GWAS can identify important SNPs and candidate genes and may also be used in molecular breeding to shorten the period of poultry breeding [13]. At present, GWAS has been widely used to study the growth, reproduction and genetic structure of various species [14].

In this study, we selected a population of Rhode Island Red (RIR) chickens to determine the phenotype and heritability of the TWC. A GWAS of the whole genome was performed through the second generation, and high-throughput sequencing was conducted to obtain candidate genes that significantly affect the TWC. The findings should provide insights for domestic poultry breeding, and the identified molecular markers may assist in molecular breeding programs.

## Materials and methods

### Experimental animals

In this study, we selected 754 eggs from the RIR population to determine the water content of the yolks, and these eggs were randomly selected during the sampling process. In this experiment, all hens were raised in separate cages under the same conditions, and they were fed the same feed throughout the experiment. From 159 hens, blood sampling was performed from the vein under the wing, and the samples were used for DNA extraction.

### Phenotype measurement

The pretreatment procedure for hot gel eggs was as follows: at one minute after a pot of water had come to a boil, the eggs were added and allowed to boil for five minutes, and then three minutes after turning off the flame, the eggs were removed. Whole yolks were then separated from the egg white and prepared for the experiment. We accurately weighed 2.0 g of gel sample from each egg and recorded it as M_1_. The sample was placed into an aluminum box, dried in an oven to a constant weight, and recorded as M_2_. The formula for calculating the water content was as follows: $\text{w}\text{a}\text{t}\text{e}\text{r} \text{c}\text{o}\text{n}\text{t}\text{e}\text{n}\text{t}/\text{\%}=\frac{\text{M}1-\text{M}2}{\text{M}1}\ast 100$. This process was repeated three times for each sample.

### Heritability

DMU software (Version: 6 swMATH, Berlin, Germany) was used to estimate the variance and covariance components. An animal threshold model was used to analyze the heritability of TWC.

The animal model was constructed as follows:

$$y = X{\rm{ }}\beta + Za + e$$

where y represents the vector of phenotypic value, β represents the vector of “fixed” effects, a represents the vector of random additive genetic effects of all individuals, e represents the vector of random residuals, and X and Z are appropriate correlation matrices. The Gibbs sampling module included in the DMU software package was used to analyze the animal threshold [15], and the DMUAI module in the DMU program was used to analyze the classical animal model using the average information limited maximum likelihood algorithm [16].

### High flux sequencing and quality control

We isolated individual genomic DNA from blood samples by the classical phenol chloroform method. DNA purity was assessed using a NanoDrop 2000 spectrophotometer (Thermo Fisher Scientific Inc., Waltham, MA, USA) and the A260/280 ratio. The whole genome was sequenced using the T7 platform of the Huada Company. Quality control was performed using Plink v1.9 [17], and the quality control standard included retaining SNPs with an allele frequency ≥ 1% and genotyping rate ≥ 98%. The individual quality control level was based on eliminating individuals whose genotype deletion rate was > 5%. SNPs in Hardy Weinberg equilibrium with P < 10 ^− 6^ were excluded. After filtration, 159 chickens were retained for further analysis.

### Population structure analysis

Before the GWAS, we first evaluated the population structure through a principal component analysis (PCA) implemented in PLINK 1.9 [18], which determined the overall structure and generate eigenvectors and eigenvalues. The plink ‘-- indep pairwise 25 5 0.2’ command was used to retain the relatively dependent SNPs. The PCA results were visualized using the “ggplot2” package in R studio. A PCA diagram was created using the first two principal components as horizontal and vertical coordinates.

### Genome wide association study

The GWAS of TWC traits was carried out using the univariate linear mixed model in GEMMA [19]. The model was as follows:

y = W α + x β + u + ε.

where y represents the n × 1 dimensional quantitative trait phenotype value vector, W represents the n × c covariate matrix j, α represents the population structure matrix calculated by ADMIX software, x represents the marked genotype, β represents the corresponding effect of SNP, u represents the vector of random effect (its covariance structure follows the normal distribution of u ~ N (0, KVg), where K is the genome relationship matrix derived from independent SNP), Vg represents the additive variance of multiple genes, and ɛ represents the error vector. In this study, the Wald test was used as a criterion for selecting SNPs associated with metabolic efficiency traits.

The “qqman” package in R was used to generate Manhattan and quantile-quantile (Q-Q) plots [20]. The genome expansion factor (λ) was calculated with R. The traditional Bonferroni correction was too strict, which resulted in a high false negative rate and led to the exclusion of SNPs that were truly related to the traits. Therefore, simpleA was used as the independent test, and the effective number of independent tests was 8,685,008. The genome-wide significance level and genome-wide suggestive significance level were set to 5.75 × 10^− 9^ (0.05/8,685,008) and 8.24 × 10^− 8^ (0.72/8,685,008), respectively. Therefore, SNPs with P values below 8.24 × 10^− 8^ were considered and may be associated with TWC traits. A linkage disequilibrium (LD) analysis of significant SNPs was performed using the solid spin algorithm implemented in Haploview version 4.2.

### Bioinformatics analysis of candidate genes

We annotated SNPs according to the Galgal 6.0 assembly supported by the Biomart tool in the Ensembl database (http://www.ensembl.org/index.html) and then searched for the nearest genes located within 400 kb upstream or downstream of important related SNPs to identify candidate genes. Then, we searched PubMed (https://pubmed.ncbi.nlm.nih.gov) for the biological functions of these genes. To provide insights into the functional enrichment of candidate genes, we carried out Gene Ontology (GO) analyses using Metascape .

## Results

### Phenotypic basic statistics and heritability test

The descriptive statistics and heritability test results for TWC are presented in Table 1. The average value of TWC was 47.73%, the minimum value was 41.06%, and the maximum value was 57.12%. These findings show that the TWC traits varied greatly from individual to individual. The heritability of the TWC traits was 0.11, which indicates medium-low heritability. The standard error of heritability shows that heritability had statistical significance. The determination of heritability showed that the TWC traits had a genetic basis.

**Table 1:** Descriptive statistics and heritability determination of the TWC traits

	Descriptive statistics	Number
Water content	Mean(%)	47.73
	SD	1.53
	Max(%)	57.12
	Min(%)	41.06
	N^b^	754
	Heritability	0.11
	SE	0.0031

### Population structure

**Fig. 1:**
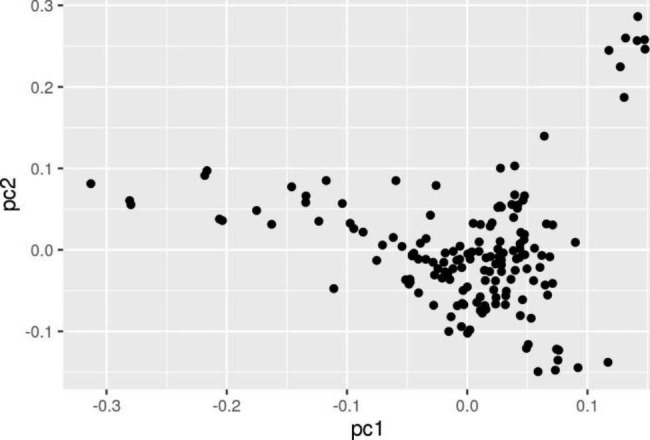
PCA plot of the population structure. The abscissa represents pc1, the ordinate represents pc2, and the point represents each individual

Genetic analyses were conducted for 159 individuals. After a series of strict quality control procedures, all samples satisfied the criteria and could be used for subsequent research. In GWAS, population stratification may lead to false positive results. The PCA showed that the RIR population was somewhat stratified (Fig. 1). Thus, we conducted a GWAS and calculated the λ value. The value of λ was 1.024, which was close to 1, indicating that population stratification was not obvious and the population could be used for the GWAS analysis.

### Genome-wide association analysis

**Fig. 2:**
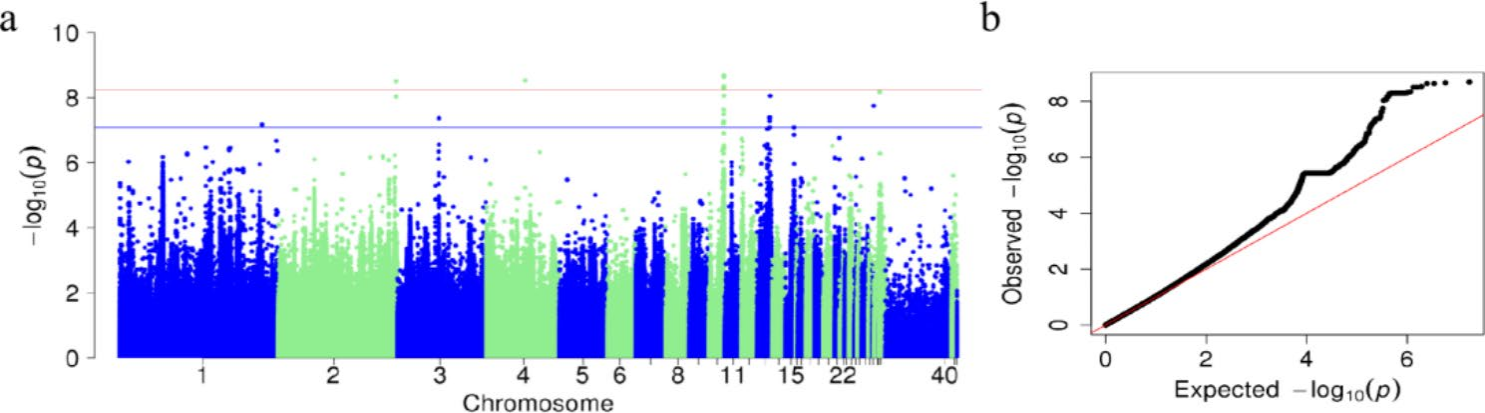
Q-Q plot and TWC traits in Manhattan Plot. The Q-Q plot shows the expected -log10 P-value (x-axis) versus the observed -log10 P-value (y-axis). In the Manhattan plot, the x-axis is the position of each SNP on the chicken chromosome (40 means Z chromosome), and the y-axis is the -log10 P-value. The horizontal red dashed line at the top represents the genome-wide significance threshold of 5.75 × 10^− 9^, and the bottom line represents the genome-wide implication threshold of 8.24 × 10^− 8^. a: Manhattan plot of TWC; b: Q-Q of TWC.

**Fig. 3:**
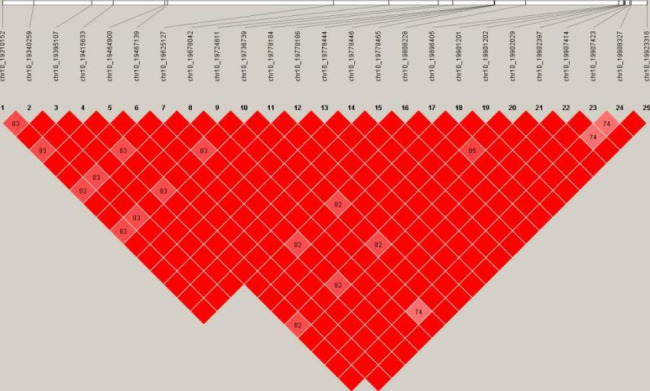
LD plots for significant SNPs. The top row represents the SNP position, and darker colors indicate greater LD intensity

The Q-Q and Manhattan plots of TWC are shown in Fig. 2. The Q-Q diagram and λ value indicated that obvious stratification did not occur in the population and the GWAS results were reliable. The Manhattan plot showed a global view of the P values (expressed in - log10 (P value)) of all SNPs. As shown in Figs. 2a and 48 SNPs were potentially related to TWC, of which 20 were significantly related and 28 were suggestively related (Table 2). Among the 48 SNPs, 25 genome-wide significant SNPs spanned a narrow 0.61 Mb region (19.31–19.92 Mb) on GGA10 (GGA for *Gallus gallus*), which represented the highest peak. Based on the analysis of significant and suggestive SNPs on GGA10 (Table 2), we found that 2 SNPs were located in the intergenic region, 7 important SNPs were located in the intron variant, 9 were located in the downstream gene variant, and 5 were located in the upstream gene variant. Based on the analysis of significant SNPs (Table 2), we found that 6 SNPs were located in the intergenic region, 17 important SNPs were located in the intron variant, 12 were located in the downstream gene variant, and 8 were located in the upstream gene variant. Significant and suggestive SNPs were mainly located on chromosomes 10 and 13. Ensembl was used to annotate the relevant SNPs, and genes located 400 kb upstream and downstream of important SNPs were searched (Table 2). The LD analysis showed that all significant SNPs within the whole genome were at high LD (Fig. 3), which increases the difficulty of identifying causal SNPs.

**Table 2:** Genome-wide SNPs around significant peaks associated with TWC traits

GGA	Position	P value	Annotation
1	177,937,248	6.64475E-08	downstream_gene_variant
1	177,937,258	6.9306E-08	downstream_gene_variant
1	177,937,260	6.9306E-08	downstream_gene_variant
2	147,631,802	3.1038E-09	intron_variant
2	147,631,804	3.1038E-09	intron_variant
2	147,631,810	9.27652E-09	intron_variant
3	51,450,796	4.22402E-08	intergenic_region
3	51,451,767	4.48935E-08	intergenic_region
4	48,300,318	2.93494E-09	intron_variant
10	19,310,152	6.2623E-08	upstream_gene_variant
10	19,340,259	5.72125E-08	synonymous_variant
10	19,395,107	5.02104E-09	intron_variant
10	19,415,633	5.72125E-08	intron_variant
10	19,464,900	5.02104E-09	intron_variant
10	19,467,139	5.02104E-09	intron_variant
10	19,625,127	2.33249E-08	intron_variant
10	19,678,042	5.02104E-09	3_prime_UTR_variant
10	19,724,611	2.15673E-09	downstream_gene_variant
10	19,738,739	3.29392E-08	upstream_gene_variant
10	19,778,184	5.24318E-09	downstream_gene_variant
10	19,778,186	5.24318E-09	downstream_gene_variant
10	19,778,444	5.02104E-09	downstream_gene_variant
10	19,778,446	5.02104E-09	downstream_gene_variant
10	19,778,465	5.02104E-09	downstream_gene_variant
10	19,808,228	4.82353E-09	intergenic_region
10	19,896,405	5.24106E-08	intergenic_region
10	19,901,201	5.02104E-09	upstream_gene_variant
10	19,901,202	5.02104E-09	upstream_gene_variant
10	19,902,029	2.40228E-08	upstream_gene_variant
10	19,902,397	4.53119E-09	intron_variant
10	19,907,414	2.27357E-09	downstream_gene_variant
10	19,907,423	2.27357E-09	downstream_gene_variant
10	19,908,327	8.70184E-09	downstream_gene_variant
10	19,923,316	2.03134E-09	intron_variant
13	15,861,318	4.113E-08	intron_variant
13	15,932,860	5.24339E-08	upstream_gene_variant
13	15,957,569	8.25751E-08	5_prime_UTR_variant
13	15,957,593	3.95242E-08	5_prime_UTR_premature_start_codon_gain_variant
13	15,957,601	4.113E-08	5_prime_UTR_premature_start_codon_gain_variant
13	15,957,711	4.25616E-08	upstream_gene_variant
13	15,957,727	4.113E-08	upstream_gene_variant
13	15,967,345	4.113E-08	intron_variant
13	16,509,483	8.73341E-09	intron_variant
13	16,516,890	8.80698E-09	intron_variant
15	10,768,769	8.24974E-08	intergenic_region
30	1,560,442	1.76791E-08	intergenic_region
33	902,132	6.72479E-09	intron_variant
33	902,135	6.72479E-09	intron_variant

### SNP annotation and promising genes related to TWC

**Fig. 4:**
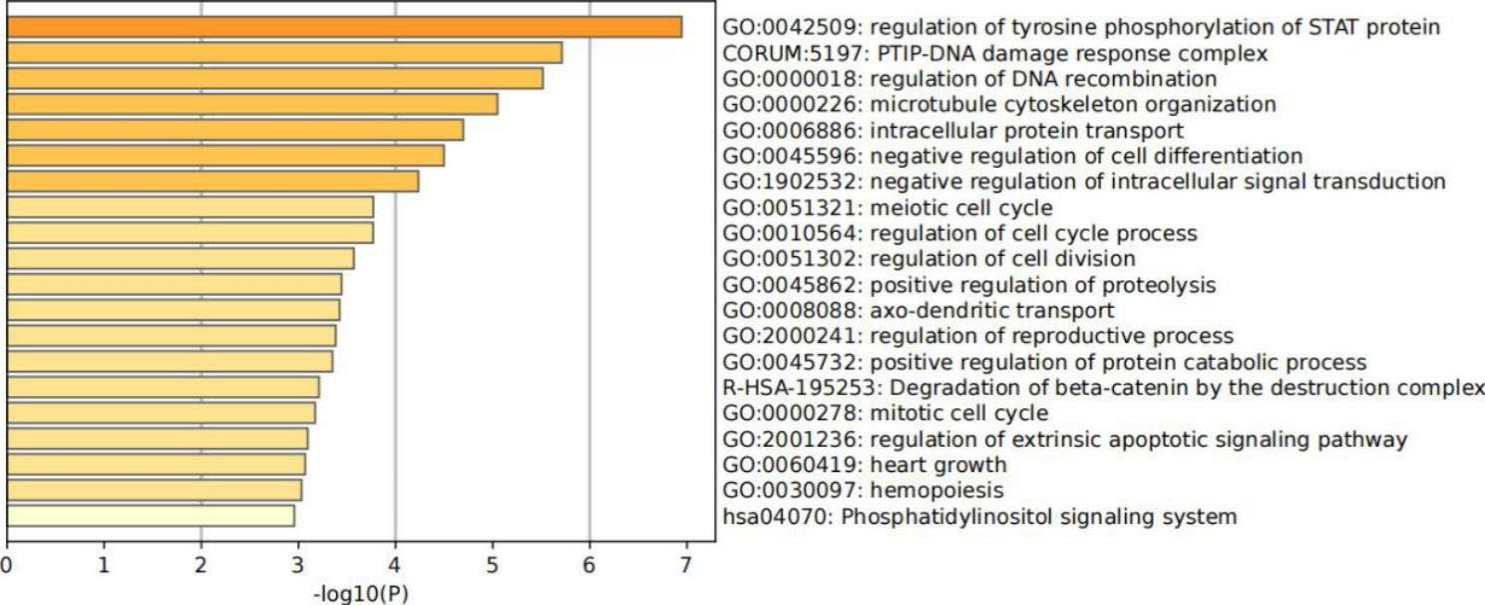
Heatmap of the top 20 candidate gene clusters and their representative enriched terms for TWC.

Using Ensembl to annotate related SNPs, we found a total of 151 potential candidate genes around the significant peaks (Table S1). We performed a GO analysis on the candidate genes (Fig. 4) and found that these genes were mainly enriched in the following terms: regulation of tyrosine phosphorylation of STAT protein (GO: 0042509), intracellular protein transport (GO: 0006886), positive regulation of protein catabolic process (GO: 0045732), and negative regulation of intracellular signal transduction (GO: 1,902,532). A search of the 151 candidate genes on PubMed revealed some important candidate genes (Table 3). *FGF9*, *PIAS1*, *FEM1B*, *NOX5*, *GLCE*, *VDAC1*, *IGFBP7*, and *THOC5* are involved in the formation and regulation of lipids [21–28]; *AP3S2*, *GNPDA1*, *HSPA4*, *AP1B1*, *CABP7*, *EEF1D*, *SYTL3*, *PPP2CA*, *SKP1*, and *UBE2B* are involved in protein folding and hydrolysis [25, 29–32]; *CSF2*, *SOWAHA*, *GDF9*, *FSTL4*, *RAPGEF6*, *PAQR5*, and *ZMAT5* are related to embryonic development and egg number laid [33–39]; *CCDC157*, *Vps33B* and *SNX9* are involved in the endocytosis cycle and transport [40–42]; *MICU2*, *ITGA11*, *WDR76*, *BLM*, *ANPEP*, *TECRL*, *EWSR1*, and *P4HA2* are associated with meat quality; and *ITGA11*, *WDR76*, *BLM* and *ANPEP* are related to meat quality water content [43–49] (Table 4).

**Table 3:** Details on the candidate genes that influence TWC traits in different ways

Gene name	GGA	Gene start (bp)	Gene end (bp)	Gene stable ID
FGF9	1	178,227,191	178,260,992	ENSGALG00010005381
PIAS1	10	19,110,135	19,162,093	ENSGALG00010017264
FEM1B	10	19,174,027	19,181,968	ENSGALG00010017354
NOX5	10	19,304,486	19,311,165	ENSGALG00010017588
GLCE	10	19,315,143	19,351,260	ENSGALG00010016452
VDAC1	13	15,634,329	15,650,729	ENSGALG00010017393
IGFBP7	4	48,670,093	48,686,869	ENSGALG00010003660
THOC5	15	11,112,860	11,124,399	ENSGALG00010000186
AP3S2	10	20,031,810	20,036,055	ENSGALG00010015942
GNPDA1	13	16,778,712	16,783,718	ENSGALG00010013300
HSPA4	13	16,753,117	16,770,088	ENSGALG00010013805
AP1B1	15	11,132,267	11,146,088	ENSGALG00010000323
CABP7	15	11,053,266	11,058,468	ENSGALG00010000312
EEF1D	2	147,784,256	147,801,359	ENSGALG00010008561
SYTL3	3	51,767,830	51,793,914	ENSGALG00010005521
PPP2CA	13	15,487,655	15,505,818	ENSGALG00010015861
SKP1	13	15,512,520	15,520,298	ENSGALG00010015876
UBE2B	13	15,460,159	15,466,303	ENSGALG00010015848
SOWAHA	13	16,632,995	16,645,991	ENSGALG00010013667
GDF9	13	16,672,170	16,675,088	ENSGALG00010013676
FSTL4	13	15,839,122	16,032,315	ENSGALG00010015972
CSF2	13	16,316,452	16,319,137	ENSGALG00010016145
RAPGEF6	13	16,081,223	16,183,984	ENSGALG00010016017
PAQR5	10	19,356,830	19,362,242	ENSGALG00010016459
ZMAT5	15	11,039,942	11,052,800	ENSGALG00010000307
VPS33B	10	20,089,069	20,096,154	ENSGALG00010000054
SNX9	3	51,372,551	51,455,373	ENSGALG00010006157
CCDC157	15	10,823,435	10,865,435	ENSGALG00010000210
MICU2	1	178,284,361	178,423,097	ENSGALG00010005396
ITGA11	10	19,182,922	19,234,018	ENSGALG00010017403
WDR76	10	19,927,476	19,933,825	ENSGALG00010016378
BLM	10	19,935,048	19,951,142	ENSGALG00010016393
ANPEP	10	20,023,232	20,028,644	ENSGALG00010015905
TECRL	4	48,587,351	48,642,364	ENSGALG00010003632
P4HA2	13	16,753,117	16,770,088	ENSGALG00010013805
EWSR1	15	11,154,604	11,175,932	ENSGALG00010000341

**Table 4:** Details on the 36 candidate genes that influence egg number traits in different ways

Impacts on reproductive traits	Genes
lipid formation and regulation	*FGF9, PIAS1, FEM1B, NOX5, GLCE, VDAC1, IGFBP7, THOC5*
protein folding and hydrolysis	*AP3S2, GNPDA1, HSPA4, AP1B1, CABP7, EEF1D, SYTL3, PPP2CA, SKP1, UBE2B*
embryonic development and egg number laid	*CSF2, SOWAHA, GDF9, FSTL4, RAPGEF6, PAQR5, ZMAT5*
endocytosis cycle and transport	*CCDC157, Vps33B and SNX9*
meat quality water content	*MICU2, ITGA11, WDR76, BLM, ANPEP, TECRL, EWSR1, P4HA2*

## Discussion

The texture of boiled egg yolk is an important factor affecting consumer acceptance and preference [3]. TWC affects the texture of egg yolk [5] and thus is an important character of egg yolk. At present, more studies have focused on the factors that influence chicken meat sensory characteristics [48], while fewer have investigated hot gel yolk characteristics. Moreover, the genetic mechanisms underlying TWC remain unclear. The lipid and protein content in the yolk also affect the water content in the yolk [50]. We measured the TWC of 754 hens’ eggs in the RIR population. The average TWC value was 47.73%, and the proportion of water in the yolk was high. The results showed that these TWC traits had significant variations (41.06–57.12%), which provides a basis for genetic research on TWC.

Here, we report the heritability of TWC traits for the first time. The heritability of TWC was 0.11, which indicates medium-low heritability. A previous study of chicken heritability showed that many traits presented medium-low heritability [51, 52]. The low heritability of TWC traits indicates that TWC is a complex trait that is affected by many factors. TWC is affected by protein and lipid contents. During the gelling process, the secondary structure of yolk protein is destroyed. The interactions between proteins and proteins, between proteins and lipids, and between proteins and water changed the spatial structure of yolk [53]. The spatial structure of protein and lipid during heating also affected water evaporation [54]. Another reason for the low heritability of TWC may be due to lack of selection for the trait in the breed. The disadvantages of this study are that the heritability of TWC traits of multiple varieties of chickens has not been tested and heritability estimates of the same character from different varieties of chicken will present deviations [55]. The determination of TWC heritability showed that TWC has a genetic basis and can be used for heritability research. Therefore, it is a heritable trait, which provides insights for poultry breeding.

To our knowledge, this is the first study to explore the TWC trait from a genetic perspective, and the results provide new ideas for the study of yolk texture. We performed a GWAS through the second generation and then conducted high-throughput sequencing. Moreover, the genetics of the yolk water content of 159 pure bred hens from the RIR population were studied. Some SNP sites and candidate genes related to the TWC trait were found. A significant peak was found on GGA10, and strong correlations were observed within the region from 19.31 to 19.92 Mb on GGA10. We found 48 SNPs that may be related to TWC (20 significantly, 28 suggestively) and identified 36 candidate genes according to their functions, which mainly affect TWC through the following three pathways: lipid and protein decomposition (*FGF9*, *PIAS1*, *FEM1B*, *NOX5*, *AP3S2*, *GNPDA1*, *HSPA4*, and *AP1B1*) [21–28], embryonic development (*CSF2*, *SOWAHA*, *GDF9*, *FSTL4*, *RAPGEF6*, *PAQR5*, and *ZMAT5*) [33–35], and texture and water content (*MICU2*, *ITGA11*, *WDR76*, *BLM*, *ANPEP, TECRL*, *EWSR1*, and *P4HA2*) [43–49]. *ITGA11*, *WDR76*, *BLM*, and *ANPEP* are located on GGA10 and likely have a significant impact on the TWC trait. ITGA11 encodes integrin subunit α 11 (same as β subunit 1) and dimerizes and forms cell surface collagen receptors that are involved in cell migration and collagen recombination and play a role in cell surface adhesion and signal transduction [56]. Studies have shown that *ITGA11* plays an important role in regulating changes of drip loss in pigs and represents an important candidate gene for regulating water content [47]. Therefore, it is reasonable to speculate that *ITGA11* has an important impact on TWC. WD40 repeat domain-containing protein 76 (WDR76) is a new Ras-regulated E3 ligase interacting protein, which controls 3T3-L1 adipocyte differentiation through HRAS stability regulation [57]. In zebrafish, *BLM* is necessary during the exponential proliferation of female GC and male meiosis, and the loss of BLM function will affect somatic and germ line cells [58]. Guo et al. found that *WDR76* and *BLM* have an important impact on the water content of duck meat [48]. ANPEP is a digestive enzyme that cleaves amino acids from the N-terminal of peptides [59] and is involved in the biochemical process of developing flavor and texture attributes [60]. ANPEP may affect the moisture of the egg yolk and subsequently the texture of the yolk. Trans-2, 3-enoyl CoA reductase-like enzyme (TECRL) located on GGA4 is a protein coding gene involved in fatty acid metabolism, fatty acid elongation, and PUFA biosynthesis, and it has significant contribution to fatty acid accumulation and metabolic regulation [61]. Moreover, TECRL is involved in the regulation of water content. P4HA2 is related to collagen synthesis, meat tenderness, and protein content [49]. THOC5 is a newly identified gene related to lipid levels and involved in high-density lipoprotein cholesterol metabolism, which affects the generation and regulation of fat [62]. These genes may play an important role in controlling TWC.

TWC is a complex trait determined by multiple genes and environmental factors. Previous studies showed that the genes *ITGA11*, *WDR76*, *BLM*, and *ANPEP* have a significant impact on water content and meat quality [48, 59]. This study also confirmed that *ITGA11*, *WDR76*, *BLM*, and *ANPE*P genes have a significant impact on TWC and likely represent the most important candidate genes for TWC. Additional research is needed before the genetics of TWC are fully understood. Moreover, the heritability and GWAS evaluations of TWC performed in this study provided helpful information for understanding the factors that influence yolk texture.

## Conclusion

In this study, the phenotype and heritability of TWC traits in the RIR population were determined, indicating that TWC is a measurable, variable, and heritable trait with a genetic basis. A GWAS of TWC revealed a series of SNP sites and candidate genes related to TWC. *ITGA11*, *WDR76*, *BLM*, and *ANPEP* may play a significant role in TWC. These results may help us better understand the molecular mechanisms underlying egg yolk water content and provide insights for poultry breeding.

## Electronic supplementary material

Below is the link to the electronic supplementary material.

**Additional file 1.**

## Data Availability

The data that support the findings of this study are openly available on the. SRA database under Bioproject accession PRJNA916197. (https://www.ncbi.nlm.nih.gov/bioproject/PRJNA916197)

## List of abbreviations

TWC: thermogelled egg yolk water content
GWAS: genome-wide association study
SNP: single nucleotide polymorphisms
RIR: Rhode Island Red

## References

[1] Liu Z, Sun C, Yan Y, Li G, Shi F, Wu G, Liu A, Yang N (2018). Genetic variations for egg quality of chickens at late laying period revealed by genome-wide association study. Sci Rep.

[2] Perini F, Cendron F, Rovelli G, Castellini C, Cassandro M, Lasagna E. Emerging Genetic Tools to Investigate Molecular Pathways Related to Heat Stress in Chickens: A Review. In: Animals., vol. 11; 2021, 46. 10.3390/ani11010046 PMC7823582 33383690

[3] Zhang R, Li X, Ma Y, Liu Y, Zhang Y, Cheng X, Ning Z. Identification of candidate genomic regions for thermogelled egg yolk traits based on a genome-wide association study.POULTRY SCI2022:102402. 10.1016/j.psj.2022.102402 PMC9850194 36610105

[4] Li J, Xu L, Su Y, Chang C, Yang Y, Gu L (2020). Flocculation behavior and gel properties of egg yolk/κ-carrageenan composite aqueous and emulsion systems: Effect of NaCl. FOOD RES INT.

[5] Li J, Li X, Wang C, Zhang M, Xu Y, Zhou B, Su Y, Yang Y (2018). Characteristics of gelling and water holding properties of hen egg white/yolk gel with NaCl addition. FOOD HYDROCOLLOID.

[6] Razi SM, Fahim H, Amirabadi S, Rashidinejad A (2023). An overview of the functional properties of egg white proteins and their application in the food industry. FOOD HYDROCOLLOID.

[7] Zhang R, Deng J, Li X, Shang W, Ning Z (2022). Research note: comparison of the texture, structure, and composition of eggs from local chinese chickens and a highly selected line of egg-type chickens and analysis of the effects of lipids on texture. Poult SCI.

[8] Kalkani A, Paraskevopoulou A, Kiosseoglou V (2007). Protein interactions and filler effects in heat-set gels based on egg. FOOD HYDROCOLLOID.

[9] Goto T, Shimamoto S, Takaya M, Sato S, Takahashi K, Nishimura K, Morii Y, Kunishige K, Ohtsuka A, Ijiri D (2021). Impact on genetic differences among various chicken breeds on free amino acid contents of egg yolk and albumen. SCI REP-UK.

[10] Franco D, Rois D, Arias A, Justo JR, Marti-Quijal FJ, Khubber S, Barba FJ, López-Pedrouso M, Manuel Lorenzo J. Effect of Breed and Diet Type on the Freshness and Quality of the Eggs: A Comparison between Mos (Indigenous Galician Breed) and Isa Brown Hens. Foods (Basel, Switzerland) 2020, 9(3):342. 10.3390/foods9030342 PMC7142747 32188038

[11] Jiang L, Liu X, Yang J, Wang H, Jiang J, Liu L, He S, Ding X, Liu J, Zhang Q (2014). Targeted resequencing of GWAS loci reveals novel genetic variants for milk production traits. BMC Genomics.

[12] Zhang GX, Fan QC, Wang JY, Zhang T, Xue Q, Shi HQ (2015). Genome-wide association study on reproductive traits in Jinghai Yellow Chicken. ANIM REPROD SCI.

[13] Eltaher S, Baenziger PS, Belamkar V, Emara HA, Nower AA, Salem KFM, Alqudah AM, Sallam A (2021). GWAS revealed effect of genotype × environment interactions for grain yield of Nebraska winter wheat. BMC Genomics.

[14] Cole JB, Wiggans GR, Ma L, Sonstegard TS, Lawlor TJ, Crooker BA, Van Tassell CP, Yang J, Wang S, Matukumalli LK (2011). Genome-wide association analysis of thirty one production, health, reproduction and body conformation traits in contemporary U.S. Holstein cows. BMC Genomics.

[15] Ødegård J, Meuwissen THE, Heringstad B, Madsen P (2010). A simple algorithm to estimate genetic variance in an animal threshold model using bayesian inference. Genet selection evolution: GSE.

[16] Li X, Nie C, Zhang Z, Wang Q, Shao P, Zhao Q, Chen Y, Wang D, Li Y, Jiao W (2018). Evaluation of genetic resistance to Salmonella Pullorum in three chicken lines. Poult SCI.

[17] Purcell S, Neale B, Todd-Brown K, Thomas L, Ferreira MAR, Bender D, Maller J, Sklar P, de Bakker PIW, Daly MJ (2007). PLINK: a Tool Set for whole-genome Association and Population-Based linkage analyses. Am J Hum Genet.

[18] William A, David JB (2009). Population structure and cryptic relatedness in Genetic Association Studies. STAT SCI.

[19] Zhou X, Stephens M (2012). Genome-wide efficient mixed-model analysis for association studies. NAT GENET.

[20] Shen M, Qu L, Ma M, Dou T, Lu J, Guo J, Hu Y, Yi G, Yuan J, Sun C et al. Genome-Wide Association Studies for Comb Traits in Chickens. PLOS ONE 2016, 11(7):e159081. 10.1371/journal.pone.0159081 PMC4948856 27427764

[21] Cui S, Li X, Li R, Zhang H, Wang Y, Li Y, Zhu J, Li Z, Lin Y. FGF1 promotes the differentiation of goat intramuscular and subcutaneous preadipocytes.ANIM BIOTECHNOL2021:1–13. 10.1080/10495398.2021.2016430 34939903

[22] Akil A, Song P, Peng J, Gondeau C, Samuel D, Gassama-Diagne A. PIAS1 Regulates Hepatitis C Virus-Induced Lipid Droplet Accumulation by Controlling Septin 9 and Microtubule Filament Assembly. In: Pathogens., vol. 10; 2021. 10.3390/pathogens10101327 PMC8537804 34684276

[23] Silva ÉF, Lopes MS, Lopes PS, Gasparino E (2019). A genome-wide association study for feed efficiency-related traits in a crossbred pig population. ANIMAL.

[24] García JG, Ansorena E, Milagro FI, Zalba G, de Miguel C. Endothelial Nox5 Expression Modulates Glucose Uptake and Lipid Accumulation in Mice Fed a High-Fat Diet and 3T3-L1 Adipocytes Treated with Glucose and Palmitic Acid. In:International Journal of Molecular Sciences., vol. 22; 2021. 10.3390/ijms22052729 PMC7962974 33800461

[25] Guan X, Zhao S, Xiang W, Jin H, Chen N, Lei C, Jia Y, Xu L. Genetic Diversity and Selective Signature in Dabieshan Cattle Revealed by Whole-Genome Resequencing. In: Biology., vol. 11; 2022. 10.3390/biology11091327 PMC9495734 36138806

[26] Yang L, Zheng X, Mo C, Li S, Liu Z, Yang G, Zhao Q, Li S, Mou C (2020). Transcriptome analysis and identification of genes associated with chicken sperm storage duration. Poult SCI.

[27] Mazloomi S, Sanoeei FM, Tayebinia H, Karimi J, Amiri I, Abbasi E, Khodadadi I (2022). The Association of mitochondrial translocator protein and voltage-dependent Anion Channel-1 in Granulosa cells with estradiol levels and Presence of immature follicles in polycystic ovary syndrome. J Reprod Infertil.

[28] Xu Z, Mei S, Zhou J, Zhang Y, Qiao M, Sun H, Li Z, Li L, Dong B, Oyelami FO et al. Genome-Wide Assessment of Runs of Homozygosity and Estimates of Genomic Inbreeding in a Chinese Composite Pig Breed.FRONT GENET2021,12. 10.3389/fgene.2021.720081 PMC8440853 34539748

[29] Vastagh C, Solymosi N, Farkas I, Liposits Z. Proestrus differentially regulates expression of Ion Channel and Calcium Homeostasis genes in GnRH neurons of mice. FRONT MOL NEUROSCI; 2019. p. 12. 10.3389/fnmol.2019.00137 PMC6554425 31213979

[30] Han J, Ma S, Liang B, Bai T, Zhao Y, Ma Y, MacHugh DE, Ma L, Jiang L. Transcriptome Profiling of Developing Ovine Fat Tail Tissue Reveals an Important Role for MTFP1 in Regulation of Adipogenesis.Frontiers in Cell and Developmental Biology2022,10. 10.3389/fcell.2022.839731 PMC8957931 35350385

[31] Zhao L, Zhang D, Li X, Zhang Y, Zhao Y, Xu D, Cheng J, Wang J, Li W, Lin C (2022). Comparative proteomics reveals genetic mechanisms of body weight in Hu sheep and Dorper sheep. J Proteom.

[32] Dai W, Wang Q, Zhao F, Liu J, Liu H (2018). Understanding the regulatory mechanisms of milk production using integrative transcriptomic and proteomic analyses: improving inefficient utilization of crop by-products as forage in dairy industry. BMC Genomics.

[33] Yurchenko AA, Daetwyler HD, Yudin N, Schnabel RD, Vander JC, Soloshenko V, Lhasaranov B, Popov R, Taylor JF, Larkin DM (2018). Scans for signatures of selection in russian cattle breed genomes reveal new candidate genes for environmental adaptation and acclimation. Sci Rep.

[34] Zhong C, Liu Z, Qiao X, Kang L, Sun Y, Jiang Y (2021). Integrated transcriptomic analysis on small yellow follicles reveals that sosondowah ankyrin repeat domain family member a inhibits chicken follicle selection. Anim Biosci.

[35] Xiang X, Huang X, Wang J, Zhang H, Zhou W, Xu C, Huang Y, Tan Y, Yin Z. Transcriptome Analysis of the Ovaries of Taihe Black-Bone Silky Fowls at Different Egg-Laying Stages. In: Genes., vol. 13; 2022. 10.3390/genes13112066 PMC9691135 36360303

[36] Lu X, Abdalla IM, Nazar M, Fan Y, Zhang Z, Wu X, Xu T, Yang Z. Genome-Wide Association Study on Reproduction-Related Body-Shape Traits of Chinese Holstein Cows.Animals (Basel)2021, 11(7). 10.3390/ani11071927 PMC8300307 34203505

[37] Sun Z, Zhang Z, Liu Y, Ren C, He X, Jiang Y, Ouyang Y, Hong Q, Chu M. Integrated Analysis of mRNAs and Long Non-Coding RNAs Expression of Oviduct That Provides Novel Insights into the Prolificacy Mechanism of Goat (Capra hircus). In: Genes., vol. 13; 2022. 10.3390/genes13061031 PMC9222479 35741792

[38] Wang Y, Ding X, Tan Z, Xing K, Yang T, Wang Y, Sun D, Wang C (2018). Genome-wide association study for reproductive traits in a large White pig population. ANIM GENET.

[39] Zhao Q, Chen J, Zhang X, Xu Z, Lin Z, Li H, Lin W, Xie Q. Genome-Wide Association Analysis Reveals Key Genes Responsible for Egg Production of Lion Head Goose.FRONT GENET2020,10. 10.3389/fgene.2019.01391 PMC6997537 32047514

[40] Bassaganyas L, Popa SJ, Horlbeck M, Puri C, Stewart SE, Campelo F, Ashok A, Butnaru CM, Brouwers N, Heydari K (2019). New factors for protein transport identified by a genome-wide CRISPRi screen in mammalian cells. J CELL BIOL.

[41] Galmes R, Ten Brink C, Oorschot V, Veenendaal T, Jonker C, van der Sluijs P, Klumperman J (2015). Vps33B is required for delivery of endocytosed cargo to lysosomes. TRAFFIC.

[42] Soulet F, Yarar D, Leonard M, Schmid SL (2005). SNX9 regulates dynamin assembly and is required for efficient clathrin-mediated endocytosis. MOL BIOL CELL.

[43] Li Y, Chen Z, Fang Y, Cao C, Zhang Z, Pan Y, Wang Q. Runs of Homozygosity Revealed Reproductive Traits of Hu Sheep. In: Genes., vol. 13; 2022. 10.3390/genes13101848 PMC9601733 36292733

[44] Chen C, Shiue Y, Yen C, Tang P, Chang H, Lee Y (2007). Laying traits and underlying transcripts, expressed in the hypothalamus and pituitary gland, that were associated with egg production variability in chickens. Theriogenology.

[45] San J, Du Y, Wu G, Xu R, Yang J, Hu J (2021). Transcriptome analysis identifies signaling pathways related to meat quality in broiler chickens – the extracellular matrix (ECM) receptor interaction signaling pathway. Poult SCI.

[46] Wang X, Ran X, Niu X, Huang S, Li S, Wang J (2022). Whole-genome sequence analysis reveals selection signatures for important economic traits in Xiang pigs. SCI REP-UK.

[47] Zhao X, Wang C, Wang Y, Lin H, Wang H, Hu H, Wang J (2019). Comparative gene expression profiling of muscle reveals potential candidate genes affecting drip loss in pork. BMC GENET.

[48] Guo Q, Huang L, Bai H, Wang Z, Bi Y, Chen G, Jiang Y, Chang G. Genome-Wide Association Study of Potential Meat Quality Trait Loci in Ducks. In: Genes., vol. 13; 2022. 10.3390/genes13060986 PMC9222319 35741748

[49] Wen Y, Li S, Bao G, Wang J, Liu X, Hu J, Zhao F, Zhao Z, Shi B, Luo Y. Comparative Transcriptome Analysis Reveals the Mechanism Associated With Dynamic Changes in Meat Quality of the Longissimus Thoracis Muscle in Tibetan Sheep at Different Growth Stages.Frontiers in Veterinary Science2022,9. 10.3389/fvets.2022.926725 PMC9298548 35873690

[50] Xu L, Gu L, Su Y, Chang C, Wang J, Dong S, Liu Y, Yang Y, Li J (2020). Impact of thermal treatment on the rheological, microstructural, protein structures and extrusion 3D printing characteristics of egg yolk. FOOD HYDROCOLLOID.

[51] Rozempolska-Rucinska I, Zieba G, Lukaszewicz M (2013). Heritability of individual egg hatching success versus hen hatchability in layers. Poult SCI.

[52] Berthelot F, Beaumont C, Mompart F, Girard-Santosuosso O, Pardon P, Duchet-Suchaux M (1998). Estimated heritability of the resistance to cecal carrier state of Salmonella enteritidis in chickens. Poult SCI.

[53] Zhao Y, Chen Z, Li J, Xu M, Shao Y, Tu Y (2016). Formation mechanism of ovalbumin gel induced by alkali. FOOD HYDROCOLLOID.

[54] Xiang X, Liu Y, Liu Y, Wang X, Jin Y (2020). Changes in structure and flavor of egg yolk gel induced by lipid migration under heating. FOOD HYDROCOLLOID.

[55] Dou D, Shen L, Zhou J, Cao Z, Luan P, Li Y, Xiao F, Guo H, Li H, Zhang H (2022). Genome-wide association studies for growth traits in broilers. BMC Genom Data.

[56] Wu P, Wang Y, Wu Y, Jia Z, Song Y, Liang N (2019). Expression and prognostic analyses of ITGA11, ITGB4 and ITGB8 in human non-small cell lung cancer. PEERJ.

[57] Yang J, Wang F, Chen B (2021). The role of WDR76 protein in human diseases. Bosn J Basic Med Sci.

[58] Annus T, Müller D, Jezsó B, Ullaga G, Németh B, Harami GM, Orbán L, Kovács M, Varga M (2022). Bloom syndrome helicase contributes to germ line development and longevity in zebrafish. CELL DEATH DIS.

[59] Jiang S, El-Senousey HK, Fan Q, Lin X, Gou Z, Li L, Wang Y, Fouad AM, Jiang Z (2019). Effects of dietary threonine supplementation on productivity and expression of genes related to protein deposition and amino acid transportation in breeder hens of yellow-feathered chicken and their offspring. Poult SCI.

[60] Pena RN, Gallardo D, Guàrdia MD, Reixach J, Arnau J, Amills M, Quintanilla R (2013). Appearance, flavor, and texture attributes of pig dry-cured hams have a complex polygenic genomic architecture1. J ANIM SCI.

[61] Xie Y, Liu Z, Guo J, Su X, Zhao C, Zhang C, Qin Q, Dai D, Zhao Y, Wang Z et al. MicroRNA-mRNA Regulatory Networking Fine-Tunes Polyunsaturated Fatty Acid Synthesis and Metabolism in the Inner Mongolia Cashmere Goat.FRONT GENET2021,12. 10.3389/fgene.2021.649015 PMC8206643 34149800

[62] Loja-Chango R, Salazar-Pousada D, Escobar-Valdivieso GS, Ramírez-Morán C, Espinoza-Caicedo J, Pérez-López FR, Gavilanes AWD, Chedraui P (2020). Polymorphism of the THOC5 of the transcription/export multiprotein complex and its correlation with the lipid and metabolic profile in middle-aged women. GYNECOL ENDOCRINOL.

