# Supplementary material for: Identification of candidate genomic regions for egg yolk moisture content based on a genome-wide association study
Source: BMC Genomics. 2023 Mar 14;24:110. doi: 10.1186/s12864-023-09221-8 (PMC10015838; doi:10.1186/s12864-023-09221-8)
Supplement: Supplementary file 1 — Additional file 1. [file 12864_2023_9221_MOESM1_ESM.docx]

Table S1 Genome-wide SNPs associated with TWC traits

| GGA | Position | P-value | Annotation | Gene |
| --- | --- | --- | --- | --- |
| 1 | 177937248 | 6.64475E-08 | downstream_gene_variant | SGCG,FGF9,MICU2 |
| 1 | 177937258 | 6.9306E-08 | downstream_gene_variant | SGCG,FGF9,MICU3 |
| 1 | 177937260 | 6.9306E-08 | downstream_gene_variant | SGCG,FGF9,MICU4 |
| 2 | 147631802 | 3.1038E-09 | intron_variant | LY6E,LY6CLEL,TOP1MT,MAFA,ZC3H3,NAPRT,EEF1D,TIGD5,TIGD5,PYCR3,TSTA3 |
| 2 | 147631804 | 3.1038E-09 | intron_variant | LY6E,LY6CLEL,TOP1MT,MAFA,ZC3H3,NAPRT,EEF1D,TIGD5,TIGD5,PYCR3,TSTA3 |
| 2 | 147631810 | 9.27652E-09 | intron_variant | LY6E,LY6CLEL,TOP1MT,MAFA,ZC3H3,NAPRT,EEF1D,TIGD5,TIGD5,PYCR3,TSTA3 |
| 3 | 51450796 | 4.22402E-08 | intergenic_region | ARID1B,TMEM242,ZDHHC14,SNX9,SYNJ2,SERAC1,GTF2H5,TULP4,TMEM181,DYNLT1,SYTL3,EZR |
| 3 | 51451767 | 4.48935E-08 | intergenic_region | ARID1B,TMEM242,ZDHHC14,SNX9,SYNJ2,SERAC1,GTF2H5,TULP4,TMEM181,DYNLT1,SYTL3,EZR |
| 4 | 48300318 | 2.93494E-09 | intron_variant | TECRL,IGFBP7,POLR2B |
| 10 | 19310152 | 6.2623E-08 | upstream_gene_variant | IQCH,C15orf61,MAP2K5,SKOR1,gga-mir-1642,PIAS1,CALML4,gga-mir-12217,CLN6,FEM1B,gga-mir-6550,ITGA11,CORO2B,ANP32A,NOX5,GLCE,PAQR5,KIF23,TLE3,UACA,MORF4L2 |
| 10 | 19340259 | 5.72125E-08 | synonymous_variant | IQCH,C15orf61,MAP2K5,SKOR1,gga-mir-1642,PIAS1,CALML4,gga-mir-12217,CLN6,FEM1B,gga-mir-6550,ITGA11,CORO2B,ANP32A,NOX5,GLCE,PAQR5,KIF23,TLE3,UACA,MORF4L2 |
| 10 | 19395107 | 5.02104E-09 | intron_variant | MAP2K5,SKOR1,gga-mir-1642,PIAS1,CALML4,gga-mir-12217,CLN6,FEM1B,gga-mir-6550,ITGA11,CORO2B,ANP32A,NOX5,GLCE,PAQR5,KIF23,TLE3,UACA,MORF4L2,FAM96A,SPG11,EIF3J |
| 10 | 19415633 | 5.72125E-08 | intron_variant | MAP2K5,SKOR1,gga-mir-1642,PIAS1,CALML4,gga-mir-12217,CLN6,FEM1B,gga-mir-6550,ITGA11,CORO2B,ANP32A,NOX5,GLCE,PAQR5,KIF23,TLE3,UACA,MORF4L2,FAM96A,SPG11,EIF3J,CTDSPL2 |
| 10 | 19464900 | 5.02104E-09 | intron_variant | MAP2K5,SKOR1,gga-mir-1642,PIAS1,CALML4,gga-mir-12217,CLN6,FEM1B,gga-mir-6550,ITGA11,CORO2B,ANP32A,NOX5,GLCE,PAQR5,KIF23,TLE3,UACA,MORF4L2,FAM96A,SPG11,EIF3J,CTDSPL2 |
| 10 | 19467139 | 5.02104E-09 | intron_variant | MAP2K5,SKOR1,gga-mir-1642,PIAS1,CALML4,gga-mir-12217,CLN6,FEM1B,gga-mir-6550,ITGA11,CORO2B,ANP32A,NOX5,GLCE,PAQR5,KIF23,TLE3,UACA,MORF4L2,FAM96A,SPG11,EIF3J,CTDSPL2 |
| 10 | 19625127 | 2.33249E-08 | intron_variant | ITGA11,CORO2B,ANP32A,NOX5,GLCE,PAQR5,KIF23,TLE3,UACA,MORF4L2,FAM96A,SPG11,EIF3J,CTDSPL2,FRMD5,WDR76,BLM,CTSH,gga-mir-184,TMED3,GNRHR,MESP1,ANPEP |
| 10 | 19678042 | 5.02104E-09 | 3_prime_UTR_variant | ANP32A,NOX5,GLCE,PAQR5,KIF23,TLE3,UACA,MORF4L2,FAM96A,SPG11,EIF3J,CTDSPL2,FRMD5,WDR76,BLM,CTSH,gga-mir-184,TMED3,GNRHR,MESP1,ANPEP,AP3S2,ZNF710,IDH2,SEMA4B,CIB1,GDPGP1,TTLL13 |
| 10 | 19724611 | 2.15673E-09 | downstream_gene_variant | GLCE,PAQR5,KIF23,TLE3,UACA,MORF4L2,FAM96A,SPG11,EIF3J,CTDSPL2,FRMD5,WDR76,BLM,CTSH,gga-mir-184,TMED3,GNRHR,MESP1,ANPEP,AP3S2,ZNF710,IDH2,SEMA4B,CIB1,GDPGP1,TTLL13,VPS33B,PRC1,UNC45A,MAN2A2,FES |
| 10 | 19738739 | 3.29392E-08 | upstream_gene_variant | GLCE,PAQR5,KIF23,TLE3,UACA,MORF4L2,FAM96A,SPG11,EIF3J,CTDSPL2,FRMD5,WDR76,BLM,CTSH,gga-mir-184,TMED3,GNRHR,MESP1,ANPEP,AP3S2,ZNF710,IDH2,SEMA4B,CIB1,GDPGP1,TTLL13,VPS33B,PRC1,UNC45A,MAN2A2,FES,FURIN,gga-mir-12218 |
| 10 | 19778184 | 5.24318E-09 | downstream_gene_variant | KIF23,TLE3,UACA,MORF4L2,FAM96A,SPG11,EIF3J,CTDSPL2,FRMD5,WDR76,BLM,CTSH,gga-mir-184,TMED3,GNRHR,MESP1,ANPEP,AP3S2,ZNF710,IDH2,SEMA4B,CIB1,GDPGP1,TTLL13,VPS33B,PRC1,UNC45A,MAN2A2,FES,FURIN,gga-mir-12218,MFAP1,HYPK,SERINC4,SERF2,PDIA3,CKMT1B |
| 10 | 19778186 | 5.24318E-09 | downstream_gene_variant | KIF23,TLE3,UACA,MORF4L2,FAM96A,SPG11,EIF3J,CTDSPL2,FRMD5,WDR76,BLM,CTSH,gga-mir-184,TMED3,GNRHR,MESP1,ANPEP,AP3S2,ZNF710,IDH2,SEMA4B,CIB1,GDPGP1,TTLL13,VPS33B,PRC1,UNC45A,MAN2A2,FES,FURIN,gga-mir-12218,MFAP1,HYPK,SERINC4,SERF2,PDIA3,CKMT1B |
| 10 | 19778444 | 5.02104E-09 | downstream_gene_variant | KIF23,TLE3,UACA,MORF4L2,FAM96A,SPG11,EIF3J,CTDSPL2,FRMD5,WDR76,BLM,CTSH,gga-mir-184,TMED3,GNRHR,MESP1,ANPEP,AP3S2,ZNF710,IDH2,SEMA4B,CIB1,GDPGP1,TTLL13,VPS33B,PRC1,UNC45A,MAN2A2,FES,FURIN,gga-mir-12218,MFAP1,HYPK,SERINC4,SERF2,PDIA3,CKMT1B |
| 10 | 19778446 | 5.02104E-09 | downstream_gene_variant | KIF23,TLE3,UACA,MORF4L2,FAM96A,SPG11,EIF3J,CTDSPL2,FRMD5,WDR76,BLM,CTSH,gga-mir-184,TMED3,GNRHR,MESP1,ANPEP,AP3S2,ZNF710,IDH2,SEMA4B,CIB1,GDPGP1,TTLL13,VPS33B,PRC1,UNC45A,MAN2A2,FES,FURIN,gga-mir-12218,MFAP1,HYPK,SERINC4,SERF2,PDIA3,CKMT1B |
| 10 | 19778465 | 5.02104E-09 | downstream_gene_variant | KIF23,TLE3,UACA,MORF4L2,FAM96A,SPG11,EIF3J,CTDSPL2,FRMD5,WDR76,BLM,CTSH,gga-mir-184,TMED3,GNRHR,MESP1,ANPEP,AP3S2,ZNF710,IDH2,SEMA4B,CIB1,GDPGP1,TTLL13,VPS33B,PRC1,UNC45A,MAN2A2,FES,FURIN,gga-mir-12218,MFAP1,HYPK,SERINC4,SERF2,PDIA3,CKMT1B |
| 10 | 19808228 | 4.82353E-09 | intergenic_region | TLE3,UACA,MORF4L2,FAM96A,SPG11,EIF3J,CTDSPL2,FRMD5,WDR76,BLM,CTSH,gga-mir-184,TMED3,GNRHR,MESP1,ANPEP,AP3S2,ZNF710,IDH2,SEMA4B,CIB1,GDPGP1,TTLL13,VPS33B,PRC1,UNC45A,MAN2A2,FES,FURIN,gga-mir-12218,MFAP1,HYPK,SERINC4,SERF2,PDIA3,CKMT1B,PPIP5K1 |
| 10 | 19896405 | 5.24106E-08 | intergenic_region | TLE3,UACA,MORF4L2,FAM96A,SPG11,EIF3J,CTDSPL2,FRMD5,WDR76,BLM,CTSH,gga-mir-184,TMED3,GNRHR,MESP1,ANPEP,AP3S2,ZNF710,IDH2,SEMA4B,CIB1,GDPGP1,TTLL13,VPS33B,PRC1,UNC45A,MAN2A2,FES,FURIN,gga-mir-12218,MFAP1,HYPK,SERINC4,SERF2,PDIA3,CKMT1B,PPIP5K1,MAP1A,TP53BP1 |
| 10 | 19901201 | 5.02104E-09 | upstream_gene_variant | TLE3,UACA,MORF4L2,FAM96A,SPG11,EIF3J,CTDSPL2,FRMD5,WDR76,BLM,CTSH,gga-mir-184,TMED3,GNRHR,MESP1,ANPEP,AP3S2,ZNF710,IDH2,SEMA4B,CIB1,GDPGP1,TTLL13,VPS33B,PRC1,UNC45A,MAN2A2,FES,FURIN,gga-mir-12218,MFAP1,HYPK,SERINC4,SERF2,PDIA3,CKMT1B,PPIP5K1,MAP1A,TP53BP1,TUBGCP4 |
| 10 | 19901202 | 5.02104E-09 | upstream_gene_variant | TLE3,UACA,MORF4L2,FAM96A,SPG11,EIF3J,CTDSPL2,FRMD5,WDR76,BLM,CTSH,gga-mir-184,TMED3,GNRHR,MESP1,ANPEP,AP3S2,ZNF710,IDH2,SEMA4B,CIB1,GDPGP1,TTLL13,VPS33B,PRC1,UNC45A,MAN2A2,FES,FURIN,gga-mir-12218,MFAP1,HYPK,SERINC4,SERF2,PDIA3,CKMT1B,PPIP5K1,MAP1A,TP53BP1,TUBGCP4 |
| 10 | 19902029 | 2.40228E-08 | upstream_gene_variant | TLE3,UACA,MORF4L2,FAM96A,SPG11,EIF3J,CTDSPL2,FRMD5,WDR76,BLM,CTSH,gga-mir-184,TMED3,GNRHR,MESP1,ANPEP,AP3S2,ZNF710,IDH2,SEMA4B,CIB1,GDPGP1,TTLL13,VPS33B,PRC1,UNC45A,MAN2A2,FES,FURIN,gga-mir-12218,MFAP1,HYPK,SERINC4,SERF2,PDIA3,CKMT1B,PPIP5K1,MAP1A,TP53BP1,TUBGCP4 |
| 10 | 19902397 | 4.53119E-09 | intron_variant | TLE3,UACA,MORF4L2,FAM96A,SPG11,EIF3J,CTDSPL2,FRMD5,WDR76,BLM,CTSH,gga-mir-184,TMED3,GNRHR,MESP1,ANPEP,AP3S2,ZNF710,IDH2,SEMA4B,CIB1,GDPGP1,TTLL13,VPS33B,PRC1,UNC45A,MAN2A2,FES,FURIN,gga-mir-12218,MFAP1,HYPK,SERINC4,SERF2,PDIA3,CKMT1B,PPIP5K1,MAP1A,TP53BP1,TUBGCP4 |
| 10 | 19907414 | 2.27357E-09 | downstream_gene_variant | TLE3,UACA,MORF4L2,FAM96A,SPG11,EIF3J,CTDSPL2,FRMD5,WDR76,BLM,CTSH,gga-mir-184,TMED3,GNRHR,MESP1,ANPEP,AP3S2,ZNF710,IDH2,SEMA4B,CIB1,GDPGP1,TTLL13,VPS33B,PRC1,UNC45A,MAN2A2,FES,FURIN,gga-mir-12218,MFAP1,HYPK,SERINC4,SERF2,PDIA3,CKMT1B,PPIP5K1,MAP1A,TP53BP1,TUBGCP4 |
| 10 | 19907423 | 2.27357E-09 | downstream_gene_variant | TLE3,UACA,MORF4L2,FAM96A,SPG11,EIF3J,CTDSPL2,FRMD5,WDR76,BLM,CTSH,gga-mir-184,TMED3,GNRHR,MESP1,ANPEP,AP3S2,ZNF710,IDH2,SEMA4B,CIB1,GDPGP1,TTLL13,VPS33B,PRC1,UNC45A,MAN2A2,FES,FURIN,gga-mir-12218,MFAP1,HYPK,SERINC4,SERF2,PDIA3,CKMT1B,PPIP5K1,MAP1A,TP53BP1,TUBGCP4 |
| 10 | 19908327 | 8.70184E-09 | downstream_gene_variant | TLE3,UACA,MORF4L2,FAM96A,SPG11,EIF3J,CTDSPL2,FRMD5,WDR76,BLM,CTSH,gga-mir-184,TMED3,GNRHR,MESP1,ANPEP,AP3S2,ZNF710,IDH2,SEMA4B,CIB1,GDPGP1,TTLL13,VPS33B,PRC1,UNC45A,MAN2A2,FES,FURIN,gga-mir-12218,MFAP1,HYPK,SERINC4,SERF2,PDIA3,CKMT1B,PPIP5K1,MAP1A,TP53BP1,TUBGCP4 |
| 10 | 19923316 | 2.03134E-09 | intron_variant | TLE3,UACA,MORF4L2,FAM96A,SPG11,EIF3J,CTDSPL2,FRMD5,WDR76,BLM,CTSH,gga-mir-184,TMED3,GNRHR,MESP1,ANPEP,AP3S2,ZNF710,IDH2,SEMA4B,CIB1,GDPGP1,TTLL13,VPS33B,PRC1,UNC45A,MAN2A2,FES,FURIN,gga-mir-12218,MFAP1,HYPK,SERINC4,SERF2,PDIA3,CKMT1B,PPIP5K1,MAP1A,TP53BP1,TUBGCP4 |
| 13 | 15861318 | 4.113E-08 | intron_variant | UBE2B,PPP2CA,SKP1,TCF7,VDAC1,C5orf15,FSTL4,RAPGEF6,FNIP1,MEIKIN |
| 13 | 15932860 | 5.24339E-08 | upstream_gene_variant | TCF7,VDAC1,C5orf15,FSTL4,RAPGEF6,FNIP1,MEIKIN,CSF2,IL3,KK34 |
| 13 | 15957569 | 8.25751E-08 | 5_prime_UTR_variant | TCF7,VDAC1,C5orf15,FSTL4,RAPGEF6,FNIP1,MEIKIN,CSF2,IL3,KK34,P4HA2 |
| 13 | 15957593 | 3.95242E-08 | 5_prime_UTR_premature_start_codon_gain_variant | TCF7,VDAC1,C5orf15,FSTL4,RAPGEF6,FNIP1,MEIKIN,CSF2,IL3,KK34,P4HA2 |
| 13 | 15957601 | 4.113E-08 | 5_prime_UTR_premature_start_codon_gain_variant | TCF7,VDAC1,C5orf15,FSTL4,RAPGEF6,FNIP1,MEIKIN,CSF2,IL3,KK34,P4HA2 |
| 13 | 15957711 | 4.25616E-08 | upstream_gene_variant | TCF7,VDAC1,C5orf15,FSTL4,RAPGEF6,FNIP1,MEIKIN,CSF2,IL3,KK34,P4HA2 |
| 13 | 15957727 | 4.113E-08 | upstream_gene_variant | TCF7,VDAC1,C5orf15,FSTL4,RAPGEF6,FNIP1,MEIKIN,CSF2,IL3,KK34,P4HA2 |
| 13 | 15967345 | 4.113E-08 | intron_variant | TCF7,VDAC1,C5orf15,FSTL4,RAPGEF6,FNIP1,MEIKIN,CSF2,IL3,KK34,P4HA2 |
| 13 | 16509483 | 8.73341E-09 | intron_variant | RAPGEF6,FNIP1,MEIKIN,CSF2,IL3,KK34,P4HA2,PDLIM4,SLC22A4,SLC22A5,gga-mir-1609,IRF1,IL5,RAD50,IL13,IL4,KIF3A,CCNI2,SEPTIN8,SOWAHA,GDF9,UQCRQ,gga-mir-12223,LEAP2,AFF4,ZCCHC10,HSPA4,RNF14,GNPDA1,NDFIP1,SPRY4,FGF1,ARHGAP26 |
| 13 | 16516890 | 8.80698E-09 | intron_variant | RAPGEF6,FNIP1,MEIKIN,CSF2,IL3,KK34,P4HA2,PDLIM4,SLC22A4,SLC22A5,gga-mir-1609,IRF1,IL5,RAD50,IL13,IL4,KIF3A,CCNI2,SEPTIN8,SOWAHA,GDF9,UQCRQ,gga-mir-12223,LEAP2,AFF4,ZCCHC10,HSPA4,RNF14,GNPDA1,NDFIP1,SPRY4,FGF1,ARHGAP26 |
| 15 | 10768769 | 8.24974E-08 | intergenic_region | RTN4R,SLC35E4,TCN2,GAL3ST1,RNF215,CCDC157,SF3A1,TBC1D10A,CASTOR1,LIF,HORMAD2,MTMR3,ASCC2,UQCR10,ZMAT5,CABP7NF2,NIPSNAP1,THOC5,NEFH,AP1B1,SNORD125,EWSR1 |
| 30 | 1560442 | 1.76791E-08 | intergenic_region |  |
| 33 | 902132 | 6.72479E-09 | intron_variant |  |
| 33 | 902135 | 6.72479E-09 | intron_variant |  |
